# The Impact of Isocyanate Index and Filler Functionalities on the Performance of Flexible Foamed Polyurethane/Ground Tire Rubber Composites

**DOI:** 10.3390/polym14245558

**Published:** 2022-12-19

**Authors:** Adam Olszewski, Paulina Kosmela, Adam Piasecki, Mateusz Barczewski, Aleksander Hejna

**Affiliations:** 1 Department of Polymer Technology, Gdańsk University of Technology, Narutowicza 11/12, 80-233 Gdańsk, Poland; 2 Institute of Materials Engineering, Poznan University of Technology, Piotrowo 3, 61-138 Poznan, Poland; 3 Institute of Materials Technology, Poznan University of Technology, Piotrowo 3, 61-138 Poznan, Poland

**Keywords:** polyurethane foam, ground tire rubber, rubber modification, surface activation, polymer composites, cellular structure, thermomechanical performance

## Abstract

The structure and performance of polyurethane (PU) foams are strongly driven by the stoichiometry of the polyaddition reaction, quantitatively described by the isocyanate index. It determines the balance between isocyanate and hydroxyl groups in the reacting system and is affected by the introduction of additional functionalities originated, e.g., from applied fillers. Nevertheless, this issue is hardly taken into account in research works. Herein, the structure and performance of PU/ground tire rubber (GTR) composites differing in their isocyanate index (from 0.8 to 1.2) and prepared with and without considering the GTR functionalities in formulation development were investigated. Incorporating GTR into the PU matrix led to a reduction in average cell diameter (from 2 to 30% depending on the isocyanate index) compared to unfilled foams. However, formulation adjustments did not show a significant impact on cellular structure. The only decrease in open cell content was noted, from 10% for the 0.9 index to 40% for 1.2. Such changes were related to the increasing strength of the PU cellular structure able to maintain inside the increasing amount of carbon dioxide. On the other hand, considering hydroxyl values of GTR noticeably affected the thermomechanical performance of composites. The shift of glass transition temperature (T_g_), even by 10 °C for 1.2 isocyanate index, enhanced the performance of materials, which was expressed in an 8–62% drop in the composite performance factor, pointing to the enhanced reinforcing effect resulting from filler incorporation. The stiffening of foams, related to the variations in PU segmental structure, also caused minor changes in the course of thermal degradation of PU/GTR composites due to the inferior thermal stability of hard segments. The obtained results provide important insights into the development of formulations of PU composites filled with materials containing reactive functional groups able to disrupt the stoichiometric balance of the polyaddition reaction.

## 1. Introduction

Polyurethanes (PU) are a group of polymer materials composed of various organic units joined by urethane (carbamate) groups [1,2]. They are produced in a polyaddition reaction involving isocyanates and compounds containing hydrogen-donating groups, mainly hydroxyls [3,4]. The reaction between isocyanate and hydroxyl groups yields the urethane group, characteristic of this group of materials [5]. Variations from the stoichiometric balance between these groups determine the occurrence of additional reactions, especially reactions of isocyanates, due to the high reactivity of isocyanate groups [6,7]. In the case of the most common PU materials, foams, the reaction between isocyanates and water applied as a chemical blowing agent is also very critical. This reaction results in the generation of carbon dioxide, which acts as a blowing agent for PU foams. Multiple research works have proven that the stoichiometry of polyaddition reaction is crucial for the performance of PU materials [8,9,10,11,12,13]. The most important are additional isocyanate reactions resulting in the formation of allophanate, urea, biuret, or isocyanurate groups, which may act as physical crosslinks affecting the mechanical and thermomechanical performance of PU materials, but also enhance their fire resistance and thermal stability [14].

In addition to the numerous applications in an unmodified form, PU materials are often applied as a matrix for polymer composites [15,16]. The incorporation of various fillers may affect the above-mentioned stoichiometric balance between isocyanate and hydroxyl groups due to the presence of often-reactive functional groups [17]. This issue is particularly important for applying organic fillers, e.g., plant-based lignocellulosic materials, which are often rich in hydroxyl or carboxyl groups [18,19,20,21,22]. Other examples of fillers that may noticeably impact the polyaddition stoichiometry are solid leather waste [23] or waste rubber particles [24]. In our previous works [25,26], we reported the disturbances in the cellular structure and performance of PU foams resulting from chemical interactions between polyurethane matrix and ground tire rubber (GTR) particles. Similar effects have been noted by other researchers [27,28]. Ground tire rubber originates from the material recycling of post-consumer car tires, which are shredded under oxidative conditions, leading to the formation of oxygen-containing functional groups able to react with isocyanates [29,30]. This is elaborated upon in [31,32,33]. Such treatments are aimed at the surface development of GTR particles, either due to the increased roughness or generation of functional groups [34]. As a result, the incorporation of modified GTR particles significantly affects the stoichiometry of PU formation reactions.

The impact of additional functionalities originating from applied fillers is especially noticeable for composites based on flexible PU foams. Contrary to the rigid foams, they are obtained with lower values of the isocyanate index, quantifying the balance between isocyanate and hydroxyl groups. The values of the isocyanate index for rigid foams often exceed 2.0, while for flexible foams, they are often around 1.0 [35,36,37,38,39,40]. As a result, the partial attraction of isocyanate groups is more noticeable for flexible PUs, for which even small filler loading may affect stoichiometry. Previously [41], we investigated the impact of hydrogen peroxide and potassium permanganate treatment of GTR on the structure and performance of flexible foamed PU/GTR composites. Treatment of GTR with H_2_O_2_ caused oxidation of hydroxyl groups and the generation of carboxyl sites, which were characterized by lower reactivity with isocyanates reducing the hydroxyl values. Due to only minor interference in polyaddition stoichiometry, the mechanical performance was enhanced. On the other hand, the application of KMnO_4_ drastically increased the number of hydroxyl groups on the surface of GTR particles, even quadrupling the hydroxyl value. Such a strong attraction of the isocyanate groups led to the collapse of cellular structure and significant deterioration in the mechanical performance. 

The above-mentioned results confirm the indisputably strong impact of polyaddition stoichiometry and its variations on the structure and performance of PU foams. Therefore, it is essential to consider the potential impact of fillers’ functionalities during the development of formulations for PU materials. Nevertheless, hardly any research works consider the impact of fillers on the overall isocyanate index applied during PU composites’ manufacturing. Previously [42], we evaluated the impact of GTR thermomechanical treatments causing hydroxyl value variations on the performance of PU/GTR composites prepared with and without considering them in formulation development. The presented study completes the reported results by investigating the structure and performance of flexible PU/GTR composites prepared with varying isocyanate index and similarly with or without taking into account the hydroxyl values of GTR particles.

## 2. Materials and Methods

### 2.1. Materials

Analyzed PU foams and PU/GTR composites were synthesized from two types of polyether polyols, Rokopol^®^F3000 and Rokopol^®^V700, both acquired from PCC Group (Brzeg Dolny, Poland). Both polyols are obtained by propoxylation of glycerol. They were characterized by the hydroxyl values (L_OH_) of 53–59 and 225–250 mg KOH/g and molecular weights of 3000 and 700 g/mol, respectively. Moreover, glycerol purchased from Sigma Aldrich (Poznań, Poland) was applied as an additional polyol component. It was characterized by the L_OH_ of 1800 mg KOH/g. Commercially available methylene diphenyl diisocyanate-based isocyanate component SPECFLEX NF 434, acquired from M. B. Market Ltd. (Baniocha, Poland), characterized by free isocyanate content of 29.5%, was applied. During polyurethane preparation, three catalysts were applied. The potassium acetate dissolved in monoethylene glycol, PC CAT^®^ TKA30, purchased from Performance Chemicals (Belvedere, UK), was used as a crosslinking catalyst. The 33 wt.% solution of 1,4-Diazabicyclo [2.2.2]octane in dipropylene glycol, Dabco 33LV acquired from Air Products (Allentown, PA, USA), was applied as a gelling catalyst. Dibutyltin dilaurate (DBTDL) from Sigma Aldrich (Saint Louis, MO, USA) was used as the catalyst of the polyaddition reaction. Distilled water was applied as a chemical blowing agent. Ground tire rubber obtained from Recykl S.A. (Śrem, Poland) was applied as a filler for the prepared composites. It was obtained in the process of ambient grinding of post-consumer tires (a mix of passenger cars and truck tires). The applied GTR was characterized by the L_OH_ of 61.7 mg KOH/g and the mean particle size of 0.6 mm.

### 2.2. Preparation of PU Foams and PU/GTR Composites

Polyurethane foams and composites were prepared on a laboratory scale using a single-step method with an isocyanate index varying from 0.8 to 1.2. Detailed compositions of prepared materials are summarized in Table 1. After weighing, all of the compounds (isocyanate was added last) were immediately mixed for 10 s at 1800 rpm using a mechanical mixer. Then, the reacting mixture was poured into a closed aluminum mold with dimensions of 200 × 100 × 40 mm^3^. In the case of composite foams, prior to mixing all of the compounds, GTR was mixed with polyols at 1000 rpm for 60 s using a mechanical mixer to ensure satisfactory dispersion of particles. After demolding, samples were conditioned for 24 h at room temperature.

Similarly to our previous work [42,43], different variants of composites were prepared. Unfilled foams were coded as PUx. Composites whose formulations included hydroxyl values of GTR particles were coded as C-GTRx, while those which did not take into account GTR L_OH_ were coded as N-GTRx. In the case of all samples, x stands for applied isocyanate index. Formulations were developed to provide a similar level of apparent density for all foams and composites—205 ± 6 kg/m^3^.

### 2.3. Characterization Techniques

Scanning electron microscopy (SEM) was applied to characterize the cellular structure of prepared foams and composites. Investigations were carried out using a Tescan (Brno, Czech Republic) MIRA3 microscope and Jeol USA (Peabody, MA, USA) JEE 4B vacuum evaporator, which was used for sample preparation—coating with a thin, 20 nm layer of carbon. Obtained images were analyzed with free ImageJ software.

The content of open and closed cells in the analyzed foams and composites was determined using Ultrapyc 5000 Foam gas pycnometer from Anton Paar (Graz, Austria). Samples were analyzed in a nitrogen atmosphere, using a gas pressure of 3.0 psi and a temperature of 20 °C. Foams and composites were analyzed using 45 cm^3^ cell size in the corrected mode.

The thermal insulation performance of prepared foams and composites was evaluated using Netzsch HFM 446 heat flow meter from Netzsch (Selb, Germany). Thermal conductivity coefficient (λ) was measured for 200 × 100 × 40 cm^3^ rectangular samples in the temperature range of 1–19 °C (average measurement temperature of 10 °C) within seven days from foams’ manufacturing.

The foams’ and composites’ thermomechanical performance was analyzed using dynamic mechanical analysis (DMA). The Q800 DMA instrument from TA Instruments (New Castle, DE, USA) was applied. Rectangular samples with dimensions of 17 mm × 12 mm × 4 mm were analyzed in single cantilever mode at the temperature range from −130 to 100 °C, with a heating rate of 4 °C/min.

The thermal stability of analyzed samples was evaluated with thermogravimetric analysis (TGA). The Netzsch TG 209F3 analyzed from Netzsch (Selb, Germany) was employed. Samples were heated under a nitrogen atmosphere from 30 to 800 °C at the heating rate of 10 °C/min. The approximate sample weight was 10 mg. Open ceramic crucibles were applied.

## 3. Results

### 3.1. Cellular Structure of Prepared PU Foams and PU/GTR Composites

**Figure 1:**
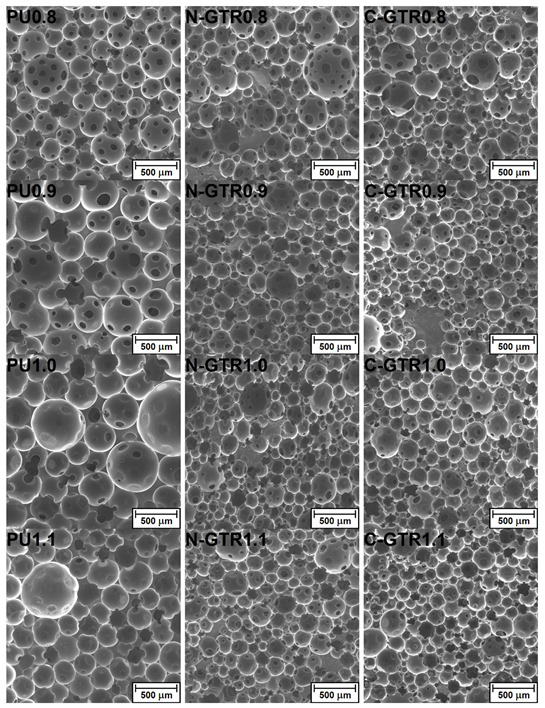

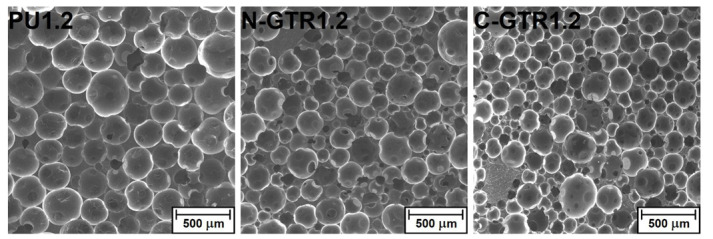
SEM images showing the cellular structure of prepared PU foams and PU/GTR composites.

Figure 1 presents the cellular structure of investigated PU foams and PU/GTR composites determined with scanning electron microscopy. Moreover, the quantitative results of the analysis of obtained images are presented in Table 2. As reported in our previous work dealing with the impact of stoichiometry on the performance of flexible PU foams [5] the biggest cell sizes for unfilled foams were noted for an isocyanate index of 1.00. Such an effect was ascribed to the balance between the amount of carbon dioxide generated during the reaction of isocyanates with water and the strength of PU cellular structure enhanced by the increasing share of isocyanate. For the isocyanate index of 1.00, the PU framework was not strong enough to keep generated CO_2_ inside small cells. When the isocyanate index exceeded unity, the isocyanate was present in excess in the system, enabling additional crosslinking reactions and strengthening the PU structure.

On the other hand, for composite foams prepared with or without considering the L_OH_ of GTR particles, the lowest average cell diameters were noted for an isocyanate index of 1.00. Such an inversion of dependence could be associated with incorporating solid particles, which could physically and chemically interact with the PU matrix during polymerization. The introduction of solid particles into the PU system increases the viscosity of reacting mixture and reduces structural homogeneity [44]. Solid GTR particles may also act as nucleating agents, increasing the number of pores and reducing their average size [45]. Considering chemical interactions, GTR particles can react with isocyanates present in the PU system due to the presence of the functional groups on their surface, mainly hydroxyls [46]. Differences between cell diameters of foams prepared with and without taking into account the L_OH_ of GTR are associated with the amount of isocyanate in the system. When hydroxyl values of GTR particles were considered, the formulations of foams were richer in isocyanate, which affected the strength of PU cellular structure, e.g., due to additional crosslinking [47]. Ideally, additional isocyanate included in formulations should react with GTR particles. However, as reported in our previous work [43], such an effect is not observed. Based on the comprehensive mechanical analysis, between 23 and 57% of additional isocyanate is consumed by GTR functional groups, and the remainder takes part in reactions leading to allophanate and biuret groups enhancing the crosslink density and strength of the foams’ structure.

As mentioned above, the dependence of the isocyanate index and the average cell size is related to the balance between the amount of CO_2_ generated and the strengthening of the PU structure. Presented results suggest that for unfilled PU foams, the impact of enhanced gas generation overpowers the strength of the cellular structure, so for the isocyanate index of 1.00, the average cell size reaches its maximum. For PU/GTR composite foams, the effect was reversed, pointing to the beneficial impact of GTR particles related to enhanced nucleation activity, which can be represented by a higher number of pores in a specific area (Figure 1). Apparently, for a different distribution of generated CO_2_ in a higher number of pores, strengthening the cellular structure via an increase in isocyanate content was sufficient to keep the gas inside.

Table 2 also provides the shape descriptors of cellular structure, circularity, aspect ratio, and roundness. Circularity describes the resemblance of shape to the perfect circle, taking into account not only the aspect ratio between perpendicular diameters, but also the quality of the perimeter [48]. Hence, its values are noticeably lower than roundness, which is simply an antagonist of aspect ratio. The circularity of cells is noticeably lower for PU/GTR composite foams, indicating that cells are frayed at the perimeter, e.g., due to the closeness of GTR particles. Moreover, due to the additional nucleating induced by the presence of solid particles, in composite foams, struts often have four or more cells, while unfilled foams showed a more typical appearance with struts connecting three more circular pores [49]. Similar to circularity, the roundness values were also reduced after incorporating solid GTR particles into the foamed PU matrix. This effect was mainly attributed to the increased heterogeneity of the cellular structure induced by the higher viscosity of the reacting mixture. Similar effects were observed in different works [50,51,52]. Interestingly, the method of foam manufacturing, i.e., whether the L_OH_ of GTR was considered or not, showed hardly any impact on the shape of pores.

Another critical parameter of the cellular structure is the share of closed and open pores. It affects multiple functional properties of foams, including thermal or sound insulation performance or water uptake. It can be seen that, irrespectively of the analyzed foams’ series, either unfilled foams or composites, the open cell content was reduced by increasing the isocyanate index. It can be ascribed to the strengthening of cell walls due to the additional crosslinking reactions, which were able to keep the increasing amounts of CO_2_ inside cells [53,54]. The most gradual decrease in open cell content with an isocyanate index from 83.1 to 22.4% was observed for unfilled foams, pointing to the efficient polymerization reactions leading to PU formation.

For composites prepared without considering GTR hydroxyl values, open cell content decreased from 82.5% for the isocyanate index of 0.80 to 64.2% for the value of 1.20. Such a small decrease points to the insufficient strength of PU cell walls due to the partial attraction of isocyanate groups by hydroxyls present on the surface of GTR particles. On the other hand, taking into account the L_OH_ of GTR and the introduction of ~16% more isocyanate resulted in the strengthening of cell walls and better trapping of generated CO_2_ inside the PU structure.

The above-mentioned structural parameters show a noticeable impact on one of the essential properties of polymeric foams—the thermal conductivity coefficient [55,56]. It is also important to underline that thermal insulation performance is more critical for rigid PU foams characterized by higher closed cell contents exceeding 90%. Flexible foams are hardly applied as thermal insulation materials. Their performance may impact the overall thermal performance of buildings only to a minor extent [57,58]. In the presented case, due to the similar level of the most critical structural parameters, apparent density, and cell size, only minor variations in the λ coefficient were observed. Therefore, it can be stated that due to the similarities in the cellular structure, neither incorporation of GTR particles nor taking into account their functionalities during formulation development affected the thermal insulation performance of prepared foams significantly.

For a deeper analysis of the interactions between the particular structural parameters of the prepared foams and composites, Pearson’s correlation coefficients (PCC) were determined. Their values are presented in Table 3. The PCC values point to a strong (between 0.6 and 0.8) or very strong (between 0.8 and 1.0) correlation between particular parameters of the cellular structure [59]. The highest correlation was noted for all series between roundness and aspect ratio, which is related to their calculation method—they are antagonists. The presented PCC values confirm the significant impact of the isocyanate index on multiple structural parameters of prepared PU foams and PU/GTR composites. For all series, very strong correlations were observed for the dependence of open cell content on the isocyanate index. The PCC value was relatively high (−0.78), even when all samples were analyzed collectively. On the other hand, despite the very strong correlations between the isocyanate index and λ coefficient for particular series, cumulative analysis of all samples did not reveal a strong correlation due to the contradictory effects noted for unfilled foam and composites. Similar observations were made for the dependence between open cell content and the thermal conductivity coefficient.

### 3.2. Thermomechanical Performance of Prepared PU Foams and PU/GTR Composites

**Figure 2:**
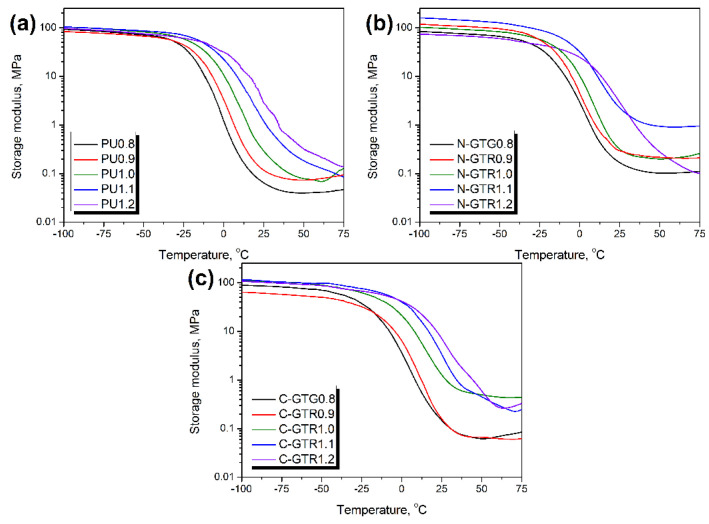
Temperature plots of storage modulus for (**a**) unfilled PU foams and PU/GTR composites prepared (**b**) without, and (**c**) with considering the hydroxyl values of GTR during formulation development.

Figure 2 presents the temperature plots of the storage modulus (E’) for unfilled PU foams and PU/GTR composites. It can be seen that, at ambient temperatures, the increase in the isocyanate index resulted in the stiffening of the foams irrespective of the applied filler or formulation adjustments. However, the most straightforward impact of the isocyanate index can be noted for unfilled foams, which can be attributed to the lack of additional effects associated with the presence of GTR particles and interface. Stiffening, expressed by the increasing E’ values, was related to the changes in foams’ chemical structures, in particular the presence of additional crosslinks resulting from the reactions of isocyanate excess [60].

**Figure 3:**
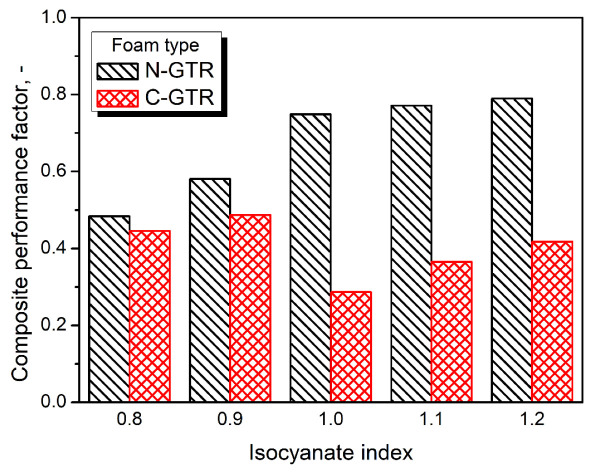
Values of composite performance factor for PU/GTR composites.

For a more detailed analysis of the stoichiometric formulation adjustments, isocyanate index, and filler functionalities on the thermomechanical performance of prepared PU/GTR composites, Figure 3 provides values of the composite performance factor (C factor). It quantifies the efficiency of the reinforcement effect resulting from particular modifications of composites according to the following Equation (1):

C = ((E’_g c_/E’_r c_)/(E’_g m_/E’_r m_)) (1)

where: E’_g_—storage modulus in the glassy state, MPa; E’_r_—storage modulus in the rubbery state, MPa; subscripts c and m refer to composite and matrix.

In the presented case, the C factor was calculated to compare the impact of the isocyanate index and formulation development approach on the performance of composites. As described in our previous works [42,61], decreasing values of the C factor point to the enhanced reinforcing effect. The composites’ performance factor values indicate that the stiffening was noted for all PU/GTR composites, confirming the data presented in Figure 2. Significantly higher values of the C factor were reported for composites prepared without formulation adjustments considering the L_OH_ of GTR particles, confirming our previous reports [42]. In the case of this group of samples, the stiffening of the PU matrix resulting from the excess of isocyanate groups in the system was limited due to the chemical interactions at the matrix/filler interface. Therefore, the generation of chemical crosslinks was inhibited, contributing to the higher mobility of PU macromolecules within the system and leading to limited stiffness enhancement. Depending on the mode of applied deformation, 23 to 57% of GTR functional groups attract free isocyanate groups present in the reacting system affecting the stoichiometric balance of polyaddition, resulting in the generation of a polyurethane matrix.

On the other hand, when hydroxyl values of GTR particles were considered during formulation development, the C factor values were significantly reduced. As mentioned above, it can be attributed to the additional isocyanate reactions generating allophanate and biuret groups. These reactions are possible due to only partial reactivity of GTR with isocyanates. Considered L_OH_ values of GTR, applied during formulation development, were determined for the more straightforward system without the competitive reactions between isocyanates and polyols. Therefore, they did not perfectly mirror the actual situation in PU/GTR composites, where reactions at the interface were not the first choice for isocyanate groups because of the steric hindrance related to the bulky structure of rubber particles.

**Figure 4:**
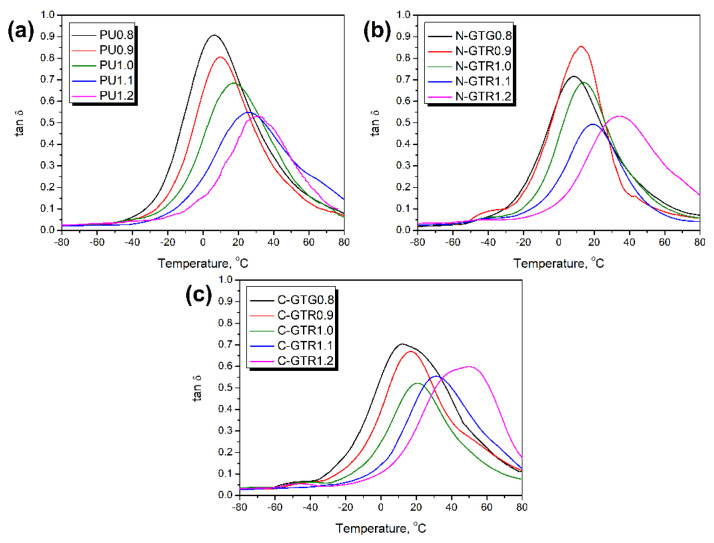
Temperature plots of loss tangent for (**a**) unfilled PU foams and PU/GTR composites prepared (**b**) without, and (**c**) with considering the hydroxyl values of GTR during formulation development.

Figure 4 shows the temperature plots of the loss tangent (tan δ) of prepared compounds and describes their ability to dissipate mechanical energy. The presented plots may provide important insights into the thermomechanical performance of the analyzed materials. The temperature position of the tan δ peak is associated with the glass transition temperature (T_g_) of the material, which in the case of PU materials, significantly depends on the stoichiometric balance between isocyanate and hydroxyl groups in the reacting system [62,63]. Glass transition in polymers is related to the transition from the hard and brittle glassy state into a viscous rubbery state, so it strongly affects the mechanical performance of materials. For all analyzed samples’ series, the increasing isocyanate index resulted in the shift of T_g_ towards higher temperatures. For unfilled foams, this was from 6.1 to 31.7 °C, while for N-GTR and C-GTR composites it was from 8.8 to 34.0 °C and from 11.5 to 44.7 °C, respectively. This behavior is typical for polyurethane materials and was repeatedly observed in other works [64,65,66]. Moreover, it confirms the results of static tensile and compressive tests reported in our previous work [43]. Considering similar values of the isocyanate index, the highest T_g_ values were noted for C-GTR composites due to the incorporation of additional isocyanate resulting from formulation adjustments. Even without applied adjustments, sample composites N-GTR showed slightly higher T_g_ values than unfilled PU foams due to the higher stiffness of GTR [67].

Besides the information about glass transition, the magnitude of tan δ provides information about the mobility of polymer macromolecules within the composite and their ability to dissipate mechanical energy. Lower peak values of tan δ indicate restricted macromolecular mobility. In the case of unfilled foams, such limitations are related to the changes in the PU chemical structure resulting from the varying isocyanate index. As mentioned above, the higher share of isocyanate in the system results in additional reactions leading to the formation of biuret and allophanate groups and enhancing structural crosslinking [5]. As presented in Figure 4, the issue was more complex in the case of PU/GTR composite foams. Although the tan δ values were generally decreasing with the rise in the isocyanate index from 0.8 to 1.2, the drop was not linear. For N-GTR composites, the lowest value was observed for the isocyanate index of 1.1, while for C-GTR composites it was 1.0. This effect may be attributed to the only partial reactivity of GTR functional groups with isocyanates due to the steric hindrance. As a result, the additional crosslinking of composites at the PU/GTR interface was not proportional to the isocyanate index values. Similar effects were noted for the storage modulus and composite performance factor.

A more comprehensive analysis of the foams’ and composites’ thermomechanical performance and the dependencies between particular parameters can be performed considering the PCC values presented in Table 4. Values indicating strong or very strong correlations are in bold. As described above, the isocyanate index showed a strong impact mainly on the T_g_ values, but also on the values of storage modulus in the rubbery state. These relationships have already been observed in multiple research works and were attributed to the changes in polyaddition stoichiometry [13,68,69,70]. Very strong dependence was also noted between E’ in the rubbery state and glass transition temperature. It was associated with the nature of the glass transition and the magnitude of the storage modulus decrease due to the loosening of the glassy structure.

### 3.3. Thermal Stability of Prepared PU Foams and PU/GTR Composites

**Figure 5:**
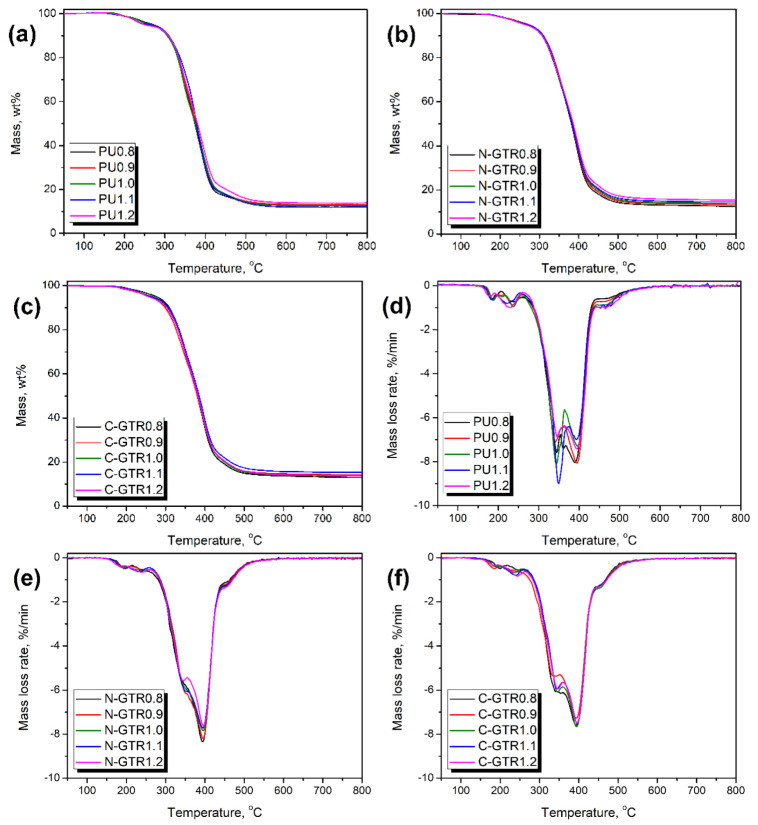
Plots of (**a**–**c**) mass loss curves and (**d**–**f**) differential thermogravimetric curves for (**a**,**d**) unfilled PU foams and PU/GTR composites prepared (**b**,**d**) without, and (**c**,**f**) with considering the hydroxyl values of GTR during formulation development.

The results of the conducted thermogravimetric analysis of prepared PU foams and PU/GTR composites are presented in Figure 5 and summarized in Table 5. It can be seen that the incorporation of GTR did not change the course of thermal decomposition, regardless of the approach for formulation development. A similar effect was noted in our previous work dealing with PU/GTR composite foams [24]. It can be attributed to the similar course of degradation of the prepared PU matrix and GTR particles. All samples showed thermal decomposition onset attributed to the 2 wt.% mass loss in the range of 210.8–223.5 °C. Taking into account the L_OH_ values of GTR in formulations hardly affected the thermal stability of the prepared composites. The stability of materials was decreased with the rise of the isocyanate index, independently of the analyzed series, due to the inferior thermal stability of the hard segments composed of urethane, allophanate, and biuret groups compared to the long macromolecular chains of polyols [71]. The first step of thermal degradation, related to the decomposition of hard segments, accounted for around 5.5, 5.0, and 6.5 wt.%, respectively, for PU, N-GTR, and C-GTR series. A slight decrease in N-GTR composites can be attributed to the reduced content of hard segments due to the partial attraction of isocyanates by GTR functionalities, which were not considered during formulation development [72]. On the other hand, the incorporation of additional isocyanate led to the excessive formation of allophanate and biuret groups that increased the hard segment content [73]. The values of the local maximum decomposition rates (T_max_) indicate that the decomposition of hard segments occurred in two steps [74]. Values of T_max1_ and T_max2_ were in the range of 182.2–203.2 °C and 223.1–245.1 °C, respectively. The observed values are in line with the literature data on PU thermal decomposition [75,76].

Noticeably more significant degradation steps were observed above 300 °C when the decomposition of the soft PU segments and GTR particles occurred. In flexible PU foams, the share of soft segments is several times higher than hard ones [77]. Typically, they decompose between 300 and 420 °C, depending on their actual structure governed by the polyols applied [78]. In the case of N-GTR and C-GTR composites, the decomposition of soft segments overlapped with the degradation of applied filler particles. The values of T_max3_ and T_max4_ correspond to the thermal decomposition of two main components of GTR—natural rubber and styrene-butadiene rubber [79]. Moreover, a small degradation step was observed above 420 °C, which can be associated with the thermolysis of residues from the previous decomposition processes [80].

## 4. Conclusions

The presented work investigated the impact of GTR functionalities and their interactions with isocyanates present in the reacting PU system on the cellular structure and thermal and thermomechanical performance of PU/GTR composites. Interestingly, the determination of the filler’s hydroxyl value does not enable the maintenance calculated values of the isocyanate index. Such an effect is related to the complexity of PU composites in terms of their chemical composition and only partial reactivity of filler functional groups with isocyanates, reduced by the steric hindrances, access to more reactive polyol hydroxyl groups and high reactivity of isocyanates with urethane, amine and urea groups. The latter reactions result in excessive stiffening of the PU structure that affects the cellular structure and mechanical performance of the materials. The obtained results confirmed our previous reports dealing with the static mechanical performance of such materials, providing important insights into the development of formulations of PU composites filled with materials containing reactive functional groups able to disrupt the stoichiometric balance of the polyaddition reaction.

## Figures and Tables

**Table 1:** Formulations applied during the preparation of PU foams.

Component	Unfilled Foams					GTR not Considered					GTR Considered				
	Content, wt.%														
F3000	35.4	34.2	33.2	32.2	31.2	29.5	28.5	27.6	26.8	26.0	28.3	27.3	26.4	25.5	24.6
V700	35.4	34.2	33.2	32.2	31.2	29.5	28.5	27.6	26.8	26.0	28.3	27.3	26.4	25.5	24.6
Glycerol	0.9	0.8	0.8	0.8	0.8	0.7	0.7	0.7	0.6	0.6	0.7	0.7	0.6	0.6	0.6
DBTDL	0.6	0.6	0.6	0.6	0.6	0.5	0.5	0.5	0.5	0.5	0.5	0.5	0.5	0.5	0.4
33LV	0.4	0.4	0.4	0.4	0.4	0.4	0.3	0.3	0.3	0.3	0.3	0.3	0.3	0.3	0.3
TKA30	0.4	0.4	0.4	0.4	0.4	0.4	0.3	0.3	0.3	0.3	0.3	0.3	0.3	0.3	0.3
Water	0.4	0.4	0.3	0.3	0.3	0.3	0.3	0.3	0.3	0.3	0.3	0.3	0.3	0.3	0.3
NF 434	26.6	28.9	31.1	33.2	35.2	22.1	24.1	25.9	27.7	29.3	24.5	26.6	28.7	30.6	32.3
GTR	-	-	-	-	-	16.7	16.7	16.7	16.7	16.7	16.7	16.7	16.7	16.7	16.7
Isocyanate index	0.8	0.9	1.0	1.1	1.2	0.8	0.9	1.0	1.1	1.2	0.8	0.9	1.0	1.1	1.2

**Table 2:** Parameters of cellular structure and thermal conductivity coefficients for prepared PU foams and PU/GTR composites.

Component	PU					N-GTR					C-GTR				
Isocyanate index	0.8	0.9	1.0	1.1	1.2	0.8	0.9	1.0	1.1	1.2	0.8	0.9	1.0	1.1	1.2
Average particle size, µm	225 ± 85	252 ± 73	274 ± 75	226 ± 73	218 ± 65	221 ± 77	200 ± 71	194 ± 61	208 ± 56	212 ± 72	222 ± 76	193 ± 72	190 ± 66	191 ± 57	208 ± 59
Circularity	0.31 ± 0.13	0.30 ± 0.11	0.31 ± 0.13	0.31 ± 0.11	0.25 ± 0.08	0.19 ± 0.12	0.28 ± 0.18	0.24 ± 0.15	0.27 ± 0.17	0.26 ± 0.16	0.19 ± 0.12	0.26 ± 0.16	0.26 ± 0.15	0.31 ± 0.17	0.29 ± 0.18
Aspect ratio	1.30 ± 0.26	1.35 ± 0.29	1.30 ± 0.24	1.27 ± 0.24	1.25 ± 0.2	1.37 ± 0.23	1.37 ± 0.24	1.39 ± 0.21	1.35 ± 0.25	1.30 ± 0.22	1.37 ± 0.25	1.38 ± 0.25	1.35 ± 0.25	1.32 ± 0.23	1.30 ± 0.23
Roundness	0.80 ± 0.13	0.77 ± 0.14	0.79 ± 0.13	0.81 ± 0.13	0.82 ± 0.11	0.75 ± 0.11	0.75 ± 0.12	0.73 ± 0.11	0.76 ± 0.12	0.79 ± 0.12	0.75 ± 0.12	0.75 ± 0.13	0.77 ± 0.13	0.78 ± 0.12	0.79 ± 0.12
Open cell content, %	83.1 ± 3.6	74.3 ± 3.3	59.9 ± 5.2	33.3 ± 4.8	22.4 ± 2.5	82.5 ± 4.1	81.7 ± 2.7	80.5 ± 1.6	76.8 ± 3.7	64.2 ± 2.2	82.9 ± 3.0	73.6 ± 2.9	66.6 ± 4.0	51.6 ± 1.9	37.9 ± 2.0
Thermal conductivity coefficient, mW/(m·K)	66.7 ± 0.8	66.8 ± 1.7	67.4 ± 1.1	70.1 ± 0.3	68.9 ± 0.5	68.7 ± 0.2	68.4 ± 0.7	68.5 ± 0.9	67.8 ± 0.9	67.9 ± 0.8	68.9 ± 1.0	69.0 ± 0.8	68.8 ± 1.6	68.0 ± 1.1	67.1 ± 0.8

**Table 3:** PCC values quantify the strength of the relationship between particular structural parameters of prepared samples.

**Structural** **Parameter**	**PU**								**N-GTR**						
	**1**	**2**	**3**	**4**	**5**	**6**	**7**		**1**	**2**	**3**	**4**	**5**	**6**	**7**
1: Isocyanate index	1.00							1	1.00						
2: Average cell size	−0.27	1.00						2	−0.15	1.00					
3: Circularity	**−0.67**	0.46	1.00					3	0.58	−0.57	1.00				
4: Aspect ratio	**−0.75**	0.59	0.53	1.00				4	**−0.74**	−0.44	−0.29	1.00			
5: Roundness	**0.66**	**−0.71**	−0.52	**−0.98**	1.00			5	**0.65**	0.51	0.28	**−0.99**	1.00		
6: Open cell content	**−0.98**	0.41	**0.64**	**0.82**	**−0.75**	1.00		6	**−0.87**	−0.21	−0.33	**0.94**	**−0.89**	1.00	
7: λ coefficient	**0.82**	−0.46	−0.27	**−0.75**	**0.71**	**−0.89**	1.00	7	**−0.87**	−0.03	**−0.67**	**0.73**	**−0.70**	**0.71**	1.00
**Structural** **Parameter**	**C-GTR**								**All Samples**						
	**1**	**2**	**3**	**4**	**5**	**6**	**7**		**1**	**2**	**3**	**4**	**5**	**6**	**7**
1: Isocyanate index	1.00							1	1.00						
2: Average cell size	−0.34	1.00						2	−0.17	1.00					
3: Circularity	**0.87**	**−0.69**	1.00					3	0.33	0.24	1.00				
4: Aspect ratio	**−0.94**	0.07	−0.71	1.00				4	**−0.61**	−0.34	−0.49	1.00			
5: Roundness	**0.97**	−0.22	**0.77**	**−0.98**	1.00			5	0.53	0.35	0.53	**−0.98**	1.00		
6: Open cell content	**−0.99**	0.23	**−0.83**	**0.96**	**−0.96**	1.00		6	**−0.78**	−0.06	−0.35	**0.82**	**−0.78**	1.00	
7: λ coefficient	**−0.89**	−0.10	**−0.62**	**0.95**	**−0.90**	**0.94**	1.00	7	0.05	−0.35	−0.40	0.05	−0.08	−0.24	1.00

**Table 4:** PCC values quantifying the strength of the relationship between the isocyanate index and particular thermomechanical parameters of prepared samples.

**Parameter**	**PU**						**N-GTR**				
	**1**	**2**	**3**	**4**	**5**		**1**	**2**	**3**	**4**	**5**
1: Isocyanate index	1.00					1	1.00				
2: E’ in glassy state	0.52	1.00				2	0.05	1.00			
3: E’ in rubbery state	**0.89**	0.27	1.00			3	**0.91**	0.16	1.00		
4: C factor	-	-	-	-		4	**0.94**	0.15	**0.75**	1.00	
5: T_g_	**0.99**	0.55	**0.91**	-	1.00	5	**0.92**	−0.23	**0.90**	**0.74**	1.00
**Parameter**	**C-GTR**						**All Samples**				
	**1**	**2**	**3**	**4**	**5**		**1**	**2**	**3**	**4**	**5**
1: Isocyanate index	1.00					1	1.00				
2: E’ in glassy state	**0.65**	1.00				2	0.29	1.00			
3: E’ in rubbery state	**0.92**	0.52	1.00			3	**0.77**	0.27	1.00		
4: C factor	−0.36	**−0.82**	−0.10	1.00		4	-	-	-	-	
5: T_g_	**0.97**	0.55	**0.98**	−0.20	1.00	5	**0.91**	0.14	**0.91**	-	1.00

**Table 5:** Results of thermogravimetric analysis of prepared PU foams and PU/GTR composites.

	Isocyanate Index	T_−2%_, °C	T_−5%_, °C	T_−10%_, °C	T_−50%_, °C	T_max1_, °C	T_max2_, °C	T_max3_, °C	T_max4_, °C	T_max5_, °C	Residue, wt.%
PU	0.8	223.5	271.4	309.2	373.8	186.9	239.1	344.5	389.7	468.4	12.51
	0.9	219.9	267.9	308.8	377.0	185.7	234.7	339.6	394.5	462.6	13.07
	1.0	221.6	266.6	305.5	374.9	187.8	236.1	343.1	395.5	461.7	12.62
	1.1	214.4	262.8	309.2	378.3	183.6	223.1	348.9	392.8	463.6	12.11
	1.2	214.0	250.0	308.1	380.3	182.2	229.5	345.4	394.0	462.8	14.12
N-GTR	0.8	214.7	270.1	306.3	381.4	195.7	245.1	345.5	394.1	460.2	12.52
	0.9	215.9	272.6	309.0	383.0	193.2	240.0	350.2	395.3	457.6	13.28
	1.0	214.7	269.4	308.6	383.4	191.4	239.7	350.2	395.4	457.6	13.94
	1.1	214.8	270.2	309.5	383.2	183.7	229.3	349.6	394.8	457.2	14.55
	1.2	211.8	263.4	306.7	385.7	185.0	239.6	344.5	396.0	452.0	15.43
C-GTR	0.8	220.9	276.7	309.2	380.5	199.7	238.5	339.8	393.2	458.1	12.95
	0.9	215.2	269.0	308.7	380.5	184.7	236.2	341.3	394.5	455.5	14.07
	1.0	214.6	266.5	305.9	382.5	203.2	238.7	346.0	394.7	455.5	13.72
	1.1	216.0	261.3	307.2	385.1	188.7	241.2	345.5	395.8	454.7	15.30
	1.2	210.8	255.5	302.8	382.7	185.1	244.5	346.4	395.6	456.2	13.76

## Data Availability

The data presented in this study are available in “The impact of isocyanate index and filler functionalities on the performance of flexible foamed polyurethane/ground tire rubber composites”.
